# A Case of Toxicity from Cannabidiol Gummy Ingestion

**DOI:** 10.7759/cureus.7688

**Published:** 2020-04-16

**Authors:** Jessica Bass, David R Linz

**Affiliations:** 1 Internal Medicine, NCH Healthcare System, Naples, USA

**Keywords:** cbd, cannabis, gummies, toxicity, overdose, cannabinoid, cannabidiol, safe, synthetic

## Abstract

A 56-year-old male with no known history of substance abuse and no known prior medical conditions presented via ambulance to the emergency department after being found by coworkers with bizarre behavior, vomiting, and slurred speech. He had legally purchased cannabidiol (CBD) gummies marketed for pain and anxiety relief at a gas station several hours prior. Vitals upon arrival were temperature 36.8 Celsius, heart rate (HR) 79, respiratory rate (RR) 12, blood pressure (BP) 113/60, and oxygen saturation (O_2_) of 84% on room air that improved upon arousal. Physical exam showed an obese man in no acute distress with a depressed level of consciousness but who awoke to painful stimuli. Neuro exam was significant for dysarthric, hypophonic speech. Labs were significant for a primary respiratory acidosis with concomitant mild lactic acid elevation, normal bicarbonate, and normal anion gap. A comprehensive urine toxicology screen including cannabis was negative. Vital signs three hours after presentation deteriorated, showing: HR 47, RR 8-12, BP 88/52, O_2_ 78%. Electrocardiogram (EKG) revealed sinus bradycardia. The patient progressively became more obtunded and required constant stimuli in order to maintain a patent airway. Non-invasive positive pressure ventilation was not administered due to persistent emesis.

The patient underwent supportive care with intravenous fluids, oxygen, anti-emetics, continuous stimulation, and close neurologic monitoring with full recovery by the following morning. Further, patient history revealed that he had consumed two packages of CBD gummies, totaling 370 mg total of CBD (serving size on the package was 30 mg). He felt the products were healthy and safe based on packaging and therefore did not believe they would have any adverse effects.

CBD is one of many cannabinoids found in marijuana and marijuana-derived products. It is generally considered safe unlike its more psychoactive counterpart, tetrahydrocannabinol (THC), which has been linked to seizures, respiratory depression, and cardiovascular complications. CBD has surged in popularity recently, being marketed in oils, capsules, and candies as a health supplement, claiming to treat a wide variety of medical conditions such as glaucoma, pain, and even having beneficial effects on cancer prevention. Most currently available studies do not look at isolated CBD nor their synthetic equivalents, and purity is not guaranteed, thus leading to unforeseen side effects and toxicities. Moreover, these compounds do not show on traditional toxicology screens, posing a diagnostic dilemma for physicians. This case of respiratory depression and cardiovascular compromise in a relatively healthy man is just one example of the importance of considering synthetic CBD toxicity in the differential diagnosis, as there is little data available for recognizing and treating this condition.

## Introduction

Cannabidiol (CBD) is a rising trend in pop-culture. It can now be found in baked goods and candies, infused into coffees, and on the shelves of stores in cosmetics and oils. Benefits have been found for specific seizure disorders such as Lennox-Gastaut syndrome and Dravet syndrome; it is also being studied for its role in neuropathic pain [1-2]. The excitement over these treatments has spurred the production of a multitude of CBD-containing products that advertise a broad spectrum of clinical benefits such as improving arthritis-related pain, potential curative effects on cancers, to an overall improvement in well-being. While these claims are unfounded, the general consensus from the World Health Organization is that CBD is well-tolerated with a good safety profile [3]. This is a case report of a 56-year-old male who experienced significant neurologic, cardiovascular, and respiratory depression due to CBD product intoxication.

## Case presentation

**Figure 1:**
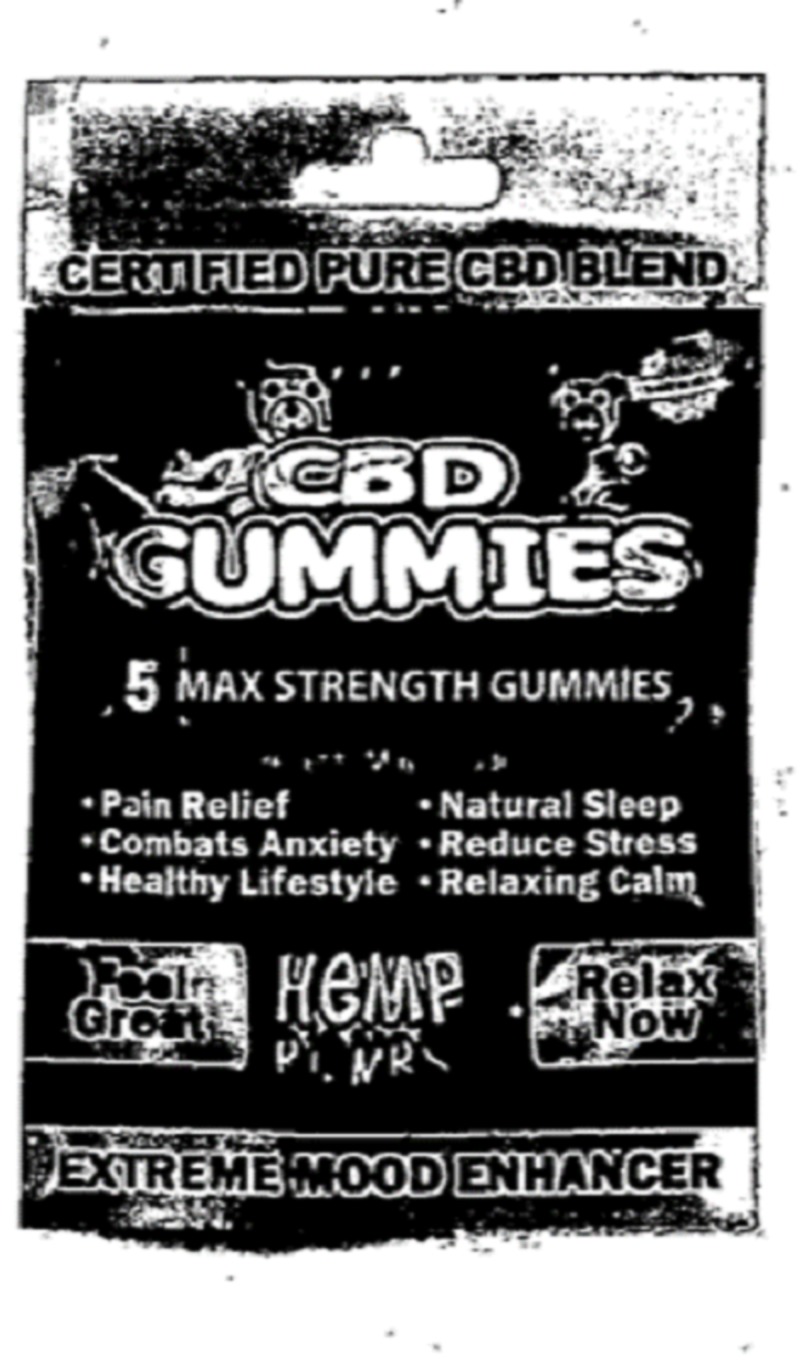
One product consumed by patient and scanned into medical record Bag of CBD gummies consumed by patient marketed as a healthy solution for pain relief. Per packaging, each gummy contained 15 mg CBD. CBD, cannabidiol

**Figure 2:**
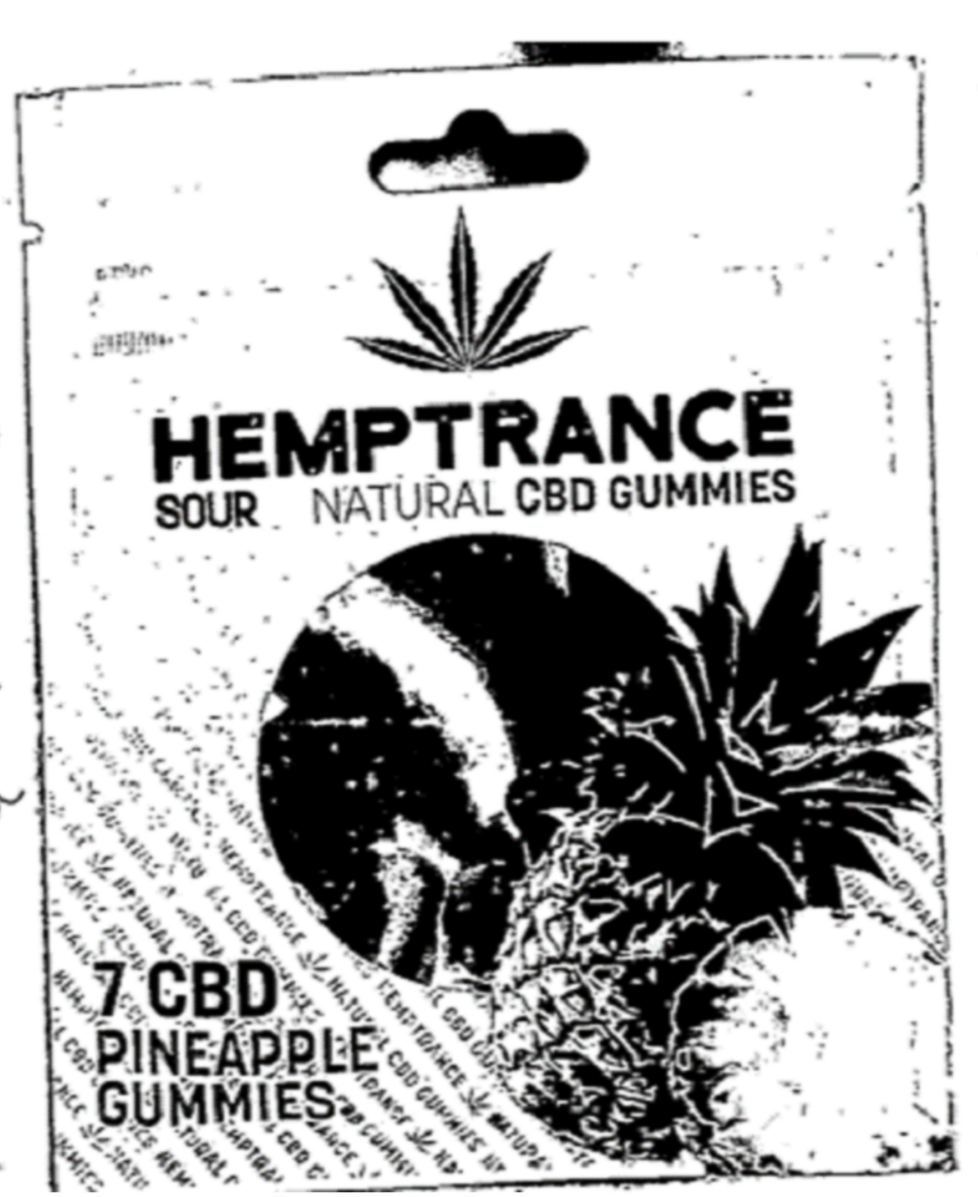
Second CBD product consumed A second bag of CBD Gummies consumed by the patient and scanned into the chart upon arrival. Product reportedly contained 50 mg CBD, 44 mg B12, and 400 IU D3 per gummy. CBD, cannabidiol

A 56-year-old male on no medications with no known history of substance abuse presented via ambulance to the emergency department after being found by coworkers with bizarre behavior, vomiting, and slurred speech. He had legally purchased “CBD gummies” marketed for pain and anxiety relief three hours prior to his presenting to the emergency department, hoping they would help relieve pain from a recent back injury (Figures 1, 2).

Upon arrival, vital signs (VS) were significant for hypoxia which did improve upon arousal. [Table 1]

**Table 1:** Vital Signs upon presentation

Initial Vital Signs	
Temperature	36.8 degrees Celsius
Heart Rate	78 beats per minute
Respiratory Rate	12 breaths per minute
Blood Pressure	113/60
Oxygen Saturation	84% on room air

Physical exam showed an obese male in no acute distress with a depressed level of consciousness, but who awoke to painful stimuli. Neuro exam was significant for dysarthric, hypophonic speech. He was also noted to have non-bilious, non-bloody emesis intermittently. Arterial blood gas at that time showed a mild acute respiratory acidosis with normal anion gap, normal bicarbonate, and a mild lactic acid elevation (Table 2).

**Table 2:** Arterial blood gas

Arterial Blood Gas	
pH	7.30
pCO_2_	50.4
pO_2_	83.3
Lactic Acid	2.4

Labs were obtained and were notable for leukocytosis and mildly elevated creatinine kinase (CK) and CKMB (Table 3). A comprehensive toxicology screen including cannabis was negative (Table 4). 

**Table 3:** Initial laboratory values WBC, white blood cell count; RBC, red blood cell count; Hb, hemoglobin; Hct: hematocrit; Na: sodium; K: potassium; Cl, chloride; CO_2_, bicarbonate; CK, creatinine kinase; CKMB, creatinine kinase-MB (H) indicates an elevated level, (L) indicates a low level

Initial Laboratory Values	
WBC	(H) 17.8
RBC	4.65
Hb	14
Hct	(L) 41.7
Platelets	208
Na	139
K	4.5
Cl	107
CO2	25
Anion Gap	7
CK	(H) 341
Ca	(L) 8.4
Serum Alcohol	<10.0
Troponin	<0.05
CKMB	(H) 4.4

**Table 4:** Urine drug screen results AMPX, amphetamines; BAR20: barbiturates; BEN, benzodiazepines; CAN 50, cannabinoids; OPI, opioids; PCP, phencyclidine; COC, cocaine; METH, methamphetamine

Urine Drug Screen	
AMPX	Negative
BAR200	Negative
BEN	Negative
CAN 50	Negative
OPI	Negative
PCP	Negative
COC	Negative
METH	Negative

**Figure 3:**
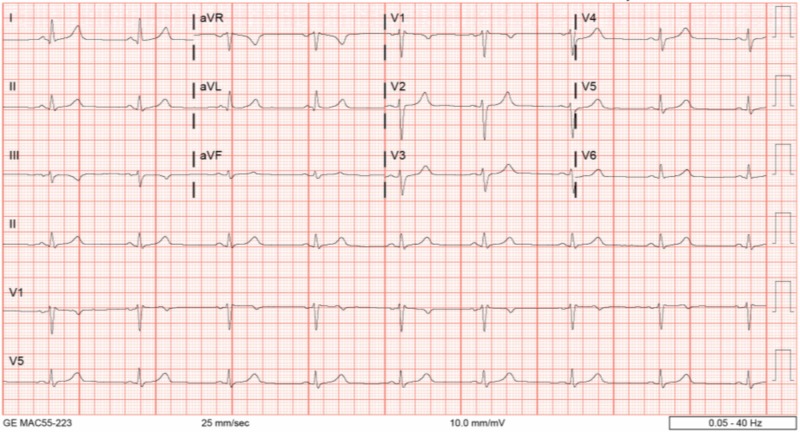
EKG demonsrating sinus bradycardia EKG, Electrocardiogram

He was admitted to the medical floor, and VS 3 hours after presentation deteriorated, showing bradycardia with a nadir HR of 47, bradypnea as low as 8, blood pressure of 88/52 mmHg, and oxygen saturation as low as 78%. EKG revealed sinus bradycardia (Figure 3). Narcan was given multiple times without improvement. The patient progressively became more obtunded but was able to maintain a patent airway with intermittent, aggressive stimuli. He was transferred to the intensive care unit (ICU) for close monitoring and possible intubation. Non-invasive positive pressure ventilation was not administered due to continued emesis.

**Figure 4:**
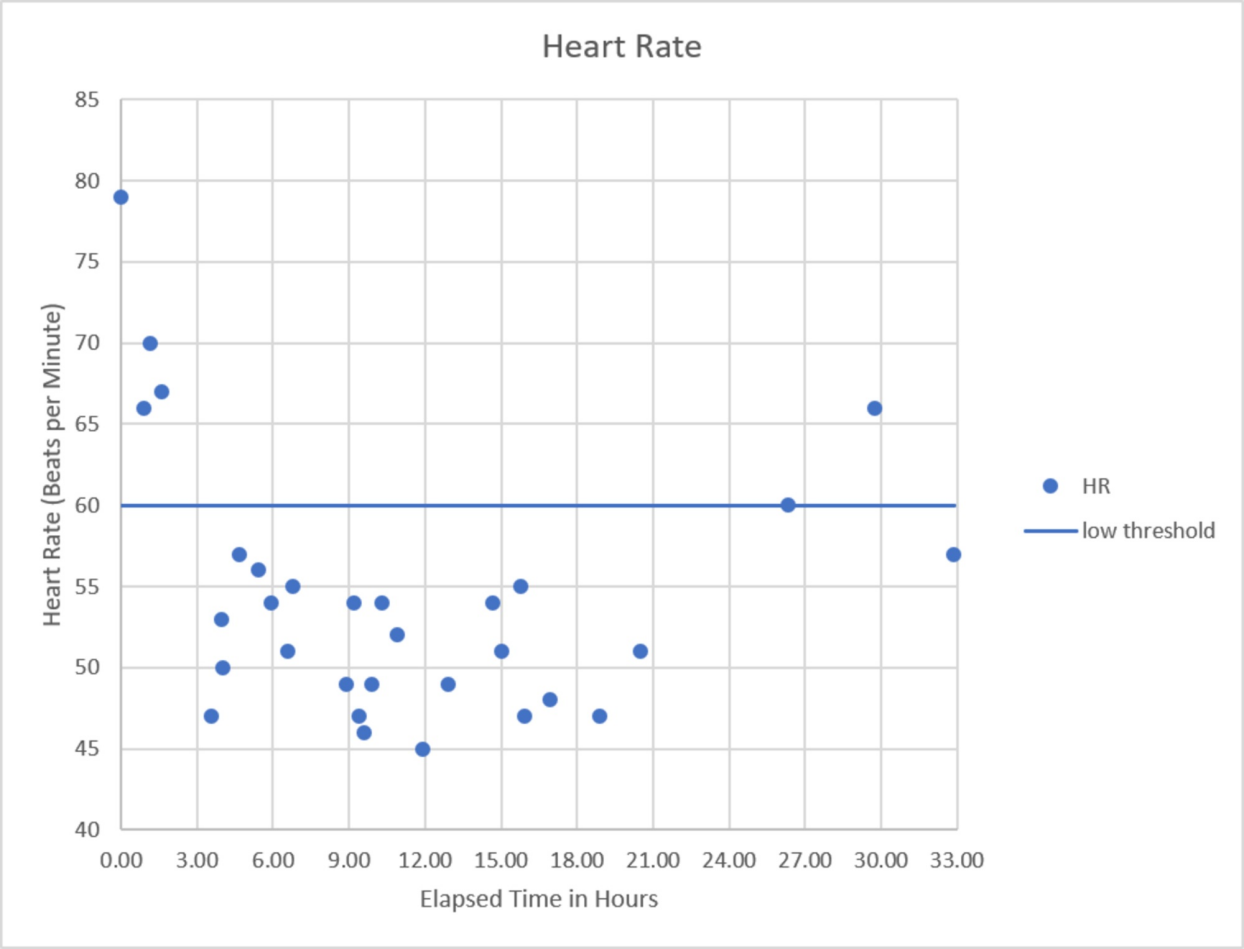
Heart rate versus time since presentation Significant bradycardia observed at approximately 3 hours after initial presentation with spontaneous recovery at approximately 26 hours.

**Figure 5:**
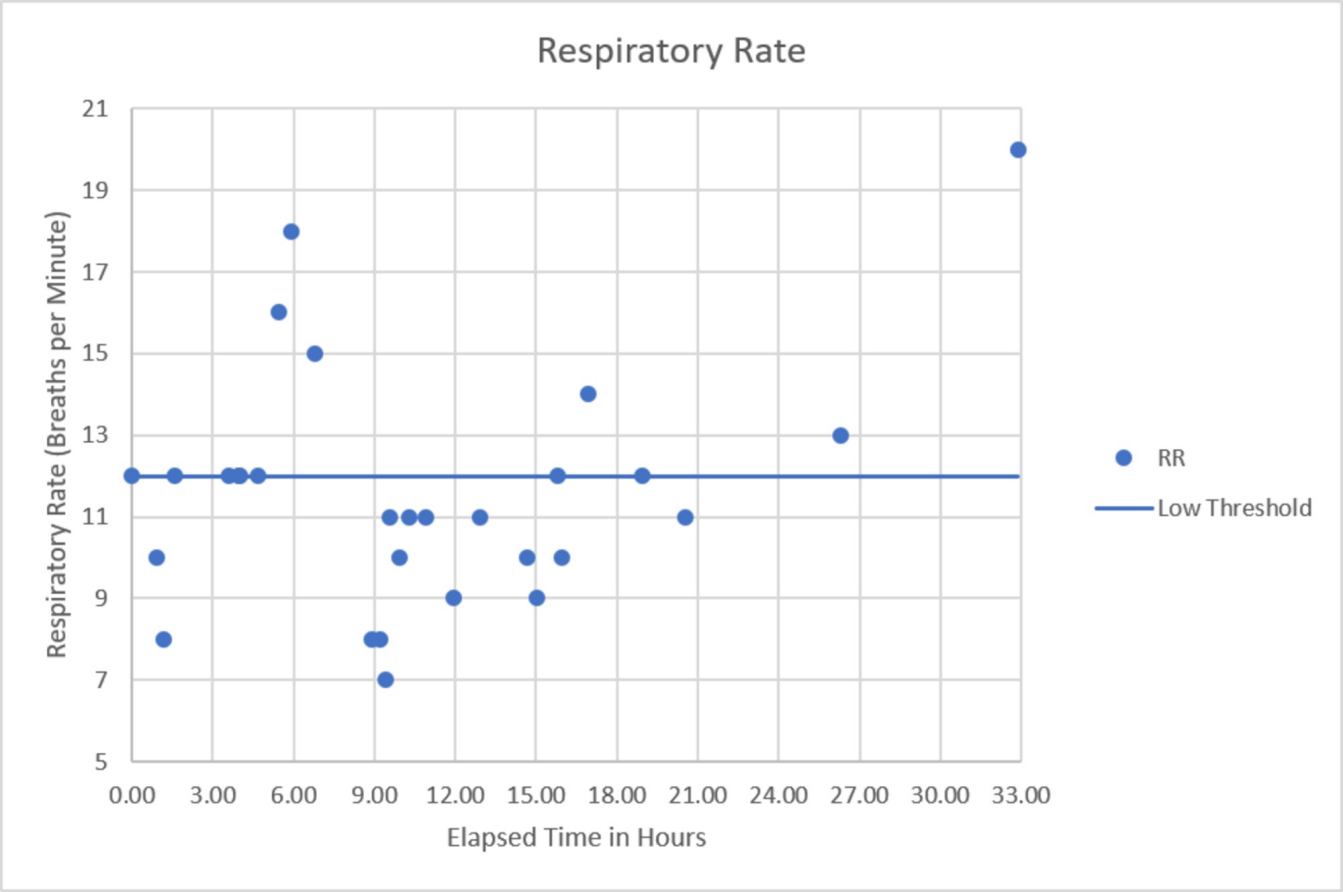
Respiratory rate versus time since presentation Upon presentation, the patient demonstrated some bradypnea which became more pronounced and persistent in the 9-21 hours after initial presentation. Again, spontaneous recovery occurred at approximately the 26-hour mark.

**Figure 6:**
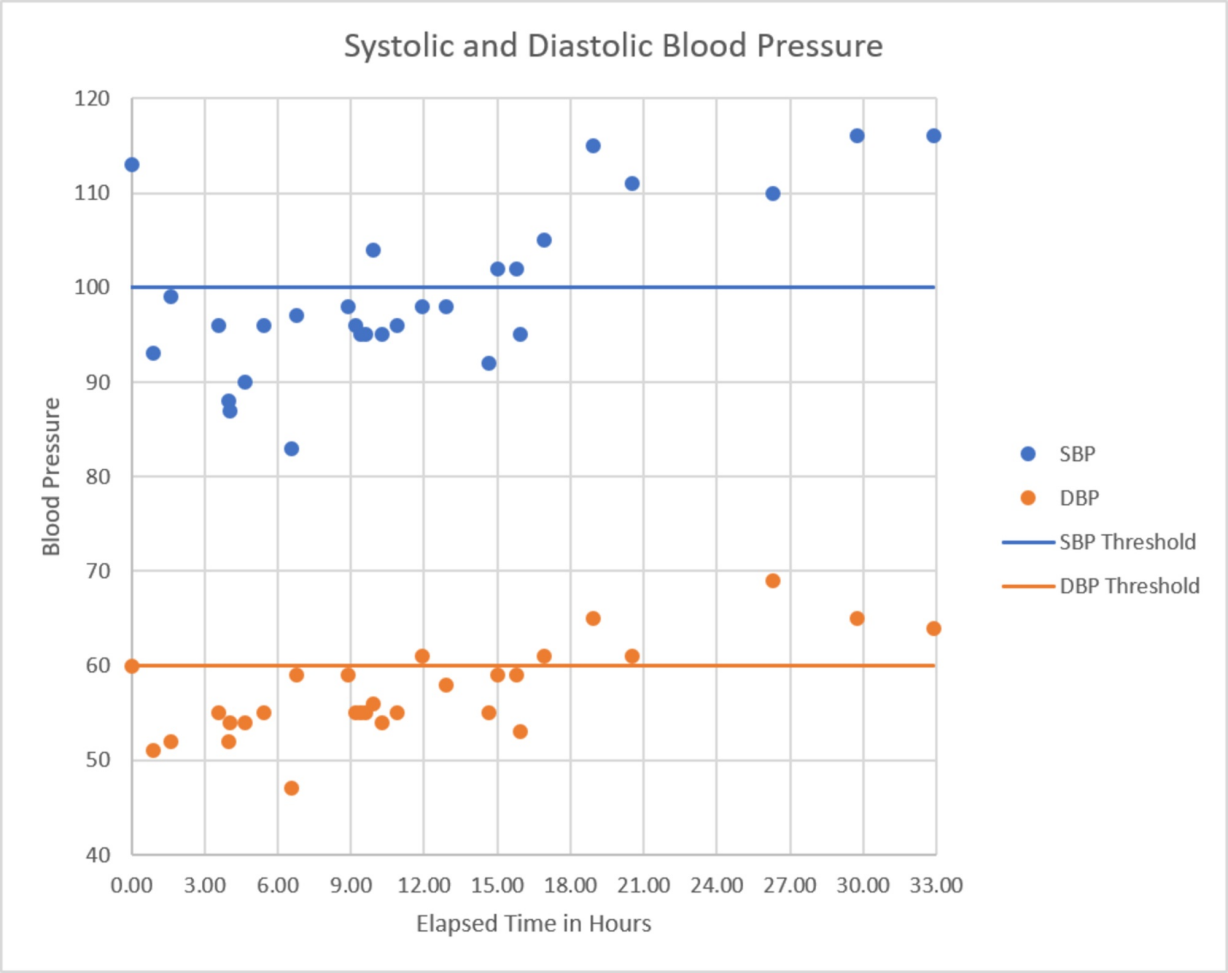
Blood pressure versus time since presentation Patient became hypotensive almost immediately after presentation which remained persistent until spontaneous recovery began to be seen at approximately 17 hours post-presentation with full recovery seen by the 26-hour period.

The patient underwent supportive care with intravenous fluids, anti-emetics, oxygen via nasal cannula, and continuous aggressive stimuli. The following day, 18 hours after admission, the patient was fully alert, oriented, and VS showed signs of improvement (Figures 4-6). The patient recalled consuming 2 entire packages of CBD gummies, totaling 370 mg of CBD (serving size on the package was 30mg). He felt the products were safe based on packaging and ate them as he would eat any other candy, not believing they would have any adverse effects.

## Discussion

This patient suffered neurologic and cardiopulmonary depression as a result of acute CBD intoxication. He was in his usual state of health prior to consuming two packs of CBD gummies. His heart rate, respiratory rate, and blood pressure dropped abruptly shortly after presentation and then experienced spontaneous recovery around 18-26 hours later without any intervention other than supportive care. This pattern of onset and recovery is consistent with toxin ingestion presumably from the CBD gummies which was the only known variable compared with the patient’s typical routine. Due to the report given by his coworkers, it was initially suspected that CBD overdose was the cause for this intoxication, of which there are no known published cases. However, given the lack of regulation and heterogeneity of over-the-counter CBD products, it is unclear which substance or substances were to blame in this patient. Similar cases are likely to be seen as these products become more commercially available.

One such case published in the literature is that of a 9-year-old boy with history of medically refractory epilepsy, diabetes insipidus, hypothyroidism, hemiplegic cerebral palsy, arachnoid cyst resection, and hypothalamic hamartoma resection who experienced profound neurologic and respiratory depression after CBD oil ingestion. The patient was being administered 1 drop of CBD oil to the gums daily but had incidental ingestion of 5 mL on the day of presentation. Upon arrival to the emergency department, he was noted to by hypothermic with depressed mental status and Glasgow coma score of 4-5 as well as decreased respiratory drive, leading to intubation. Urine screening was notable for a metabolite of THC, but testing was not readily available for CBD nor for synthetic cannabinoids. Mass spectrometry analysis of samples of the CBD oil from the same batch showed differing amounts of both CBD and THC. While the patient recovered, the exact constituent which caused the clinical findings mentioned was not able to be established due to lack of consistent formulations, purity, and potency of the commercially available products [4].

When comparing the two cases, there are certain similarities and differences which are worth mentioning. The pediatric patient consumed CBD oil, whereas the 56-year-old man ingested CBD gummies. In both scenarios, more of the CBD-labeled product was consumed than was recommended, and both patients required short-term hospitalization. In the case of the pediatric patient, testing for urine THC metabolites was also available. Neither patient had access to point-of-care testing for CBD or synthetic cannabinoids.

The CBD industry is projected to reach sales of $23 billion by 2023 [5]. This surge is likely due to the excitement behind potentially new medical breakthroughs and the desire as a culture to shift towards therapies that are more “natural.” However, most products, such as the one purchased by this patient are unregulated. Products can contain varying amounts of CBD in addition to unstudied cannabinoids, THC, or toxins such as pesticides and heavy metals. A recent study found that only 31% of 84 CBD products sold online from 31 companies were labeled correctly regarding the concentration of CBD. Additionally, THC was found in 21% of samples among other cannabinoids [6].

With the lack of a known blood level at which CBD exhibits toxic effects and the possible contamination of the product with other cannabinoids and toxins, it is difficult to know whether this is a true CBD intoxication versus toxicity from one of the constituents found in the product. Contributing to this diagnostic dilemma is the fact that the majority of these products are synthetic which allows for a larger spectrum of cannabinoids and much higher concentrations than would be found in natural sources. Additionally, synthetic products do not appear on standard toxicology screening, as was the case in this patient [7] (Oral Presentation: Bass, DO, Jessica, Linz, MD, David. Hashing Out the Unknowns of the CBD Craze. 2019 SGIM Annual Meeting; 5/11/2019).

## Conclusions

This is a case of profound neurologic, cardiac, and respiratory depression secondary to acute CBD product intoxication resulting in ICU admission. The patient's lack of substance abuse history and unintentional overdose should raise concern for physicians as more people are consuming such products. The aggressive marketing of these products paired with the lack of regulation and quality control has the potential to cause a significant negative impact on public health. Clinicians should be aware of this when prompted for advice from patients as well as when treating patients with potential intoxication. Further research into these compounds is certainly indicated and regulation may be warranted for consumer protection.

## References

[1] Chen JW, Borgelt LM, Blackmer AB (2020). Cannabidiol: a new hope for patients with Dravet or Lennox-Gastaut Syndromes. Ann Pharmacother.

[2] Überall Überall, M.A. M.A. (2020). A Review of Scientific Evidence for THC: CBD oromucosal spray (Nabiximols) in the management of chronic pain. J Pain Res.

[3] (2020). WHO. Cannabidiol (CBD) Pre-Review Report Agenda Item 5.2. https://www.who.int/medicines/access/controlled-substances/5.2_CBD.pdf.

[4] Jim Herbst, Gyen Musgrave (2020). Respiratory depression following an accidental overdose of a CBD-labeled product: a pediatric case report. J Am Pharm Assoc.

[5] Freedman DH (2020). Pop culture says CBD cures everything—here's what scientists say. Newsweek.

[6] Bonn-Miller MO, Loflin MJE, Thomas BF, Marcu JP, Hyke T, Vandrey R (2017). Labeling accuracy of cannabidiol extracts sold online. JAMA.

[7] Arntson A, Ofsa B, Lancaster D (2013). Validation of a novel immunoassay for the detection of synthetic cannabinoids and metabolites in urine specimens. J Anal Toxicol.

